# Dynamic mapping of cholera outbreak during the Yemeni Civil War, 2016–2019

**DOI:** 10.1057/s41271-022-00345-x

**Published:** 2022-05-25

**Authors:** Ryan B. Simpson, Sofia Babool, Maia C. Tarnas, Paulina M. Kaminski, Meghan A. Hartwick, Elena N. Naumova

**Affiliations:** 1 Nutrition Epidemiology and Data Science Division, Tufts University Friedman School of Nutrition Science and Policy, 150 Harrison Avenue, Boston, MA 02111 USA; 2 Neuroscience Department, The University of Texas at Dallas, Richardson, TX USA; 3 Community Health Department, Tufts University School of Arts and Sciences, Medford, MA USA

**Keywords:** Cholera, Conflict, Data alignment, Dynamic mapping, Time series, Yemen

## Abstract

Widespread destruction from the Yemeni Civil War (2014–present) triggered the world’s largest cholera outbreak. We compiled a comprehensive health dataset and created dynamic maps to demonstrate spatiotemporal changes in cholera infections and war conflicts. We aligned and merged daily, weekly, and monthly epidemiological bulletins of confirmed cholera infections and daily conflict events and fatality records to create a dataset of weekly time series for Yemen at the governorate level (subnational regions administered by governors) from 4 January 2016 through 29 December 2019. We demonstrated the use of dynamic mapping for tracing the onset and spread of infection and manmade factors that amplify the outbreak. We report curated data and visualization techniques to further uncover associations between infectious disease outbreaks and risk factors and to better coordinate humanitarian aid and relief efforts during complex emergencies.

**Supplementary Information:**

The online version contains supplementary material available at 10.1057/s41271-022-00345-x. All movies are available for download at: https://tufts.box.com/v/Simpson-2022-CholeraDynamicMap.

## Key messages

- We implore the global public health community to harmonize data collection and reporting protocols and to improve the spatiotemporal resolution of time-referenced data. This will allow external users to trace the onset, spread, and amplification of disease outbreaks.
- We recommend public health professionals use advanced data visualization techniques when investigating complex spatiotemporal patterns of infectious outbreaks.
- Our dynamic maps suggested that the cholera outbreak “travelled” from Sana’a and Sana’a City, the epicentre of the national outbreak, to surrounding governorates, including Al-Hudaydah, characterized as a persistent cluster of conflict events.

## Introduction

During complex humanitarian emergencies such as war, detailed analyses of health outcomes and related factors are difficult to conduct. High risk of disease morbidity and mortality impedes primary data collection and severely limit medical resources, personnel, and hospital bed capacity [1]. Real-time analyses are also hindered by limited available data, reduced laboratory testing capacity, and impaired or non-existent health infrastructure due to wartime events such as bombings and explosions. [2, 3]. As data collection and processing capabilities grow, the demand for use of novel approaches to integrate, examine, and present data from multiple sources to broad audiences also grows. These needs promote a new era of precision public health that prioritizes transparency in discussions of data quality, standardizes reporting of statistical methods, and uses advanced data visualization techniques to capture complex spatiotemporal patterns of diseases. Better understanding of these patterns can assist in predictive modelling for early warning outbreak systems and timely, pre-emptive medical resource and personnel mobilization to expected geographic hotspots of infection.

For example, the Yemeni Civil War (2014–present) has led to widespread destruction that has triggered the world’s largest cholera outbreak [1, 4]. Even before the war, Yemen was one of the poorest countries in the Arabian Peninsula with limited access to clean and affordable drinking water [5, 6]. High rates of malnourished and immuno-compromised persons have increased cholera infection risks particularly in infants and children [6]. Limited access to sanitation and hygiene supplies has further amplified cholera transmission, mortality, and morbidity. This especially holds true in western, mountainous, densely populated governorates, such as Sana’a and Sana’a City (nation’s capital), compared to eastern, arid, sparsely populated governorates, such as Hadhramaut and Al-Maharah [1, 3]. Frequent skirmishes between a Saudi Arabian military coalition, the Yemeni government, and the armed Houthi movement further stress health infrastructure and medical supplies, particularly after the blockade on the Port of Al-Hudaydah, which has limited imports of food, water, and medical resources like cholera vaccinations [7].

New ways of analysing and presenting data are critical for linking conflict events and fatalities with disastrous health outcomes, as with the current humanitarian emergency in Yemen. However, few studies have investigated associations between illness rates and conflict in Yemen or elsewhere perhaps due to limited publicly available data [8–12]. Some publications have used privately acquired datasets that cannot be replicated or further examined due to data usage limitations [13, 14]. Other publications have referred to humanitarian situation reports built on qualitative findings and observations from health workers [15–17]. In our prior work, we curated a weekly time series dataset of confirmed cholera infections using publicly reported World Health Organization (WHO) epidemiological bulletins from 2016 through 2019 [18]. This provided a foundation for aligning and integrating other spatiotemporal data to create publicly available datasets for further analyses.

Time-referenced conflict databases disseminate publicly available records for reuse. One example is the Armed Conflict Location and Event Data (ACLED) Project, which provides geocoded data on conflict events and fatalities for various types of violence across Asia, the Middle East, Europe, and Latin America [19–22]. Datasets compiled during wartime, such as during the Syrian Conflict (2011–present) and Somali Civil War (1991–present), have proved invaluable as sources of information for coordinating international humanitarian relief [12, 23]. Yet, these data sources reflect the deficiencies of primary data collection such as inconsistent reporting frequency, limited spatial and temporal alignment, insufficient information on reporting practices, and unclear population catchment areas, all of which create challenges in using health data for analysis, inference, and decision-making [12, 23].

Targeted efforts to standardize the preparation, reporting, and visualization of data collected over time can substantially improve the quality of public health research. Given the complexity of global health crises, dynamic maps can condense spatial and temporal information of health records into a single animated image. Dynamic maps can assist researchers in examining the seasonality of infectious disease outbreaks, the synchronization between exposure events and outbreaks, and the geographic variability of these processes [24–28]. Researchers can also investigate temporal and spatial relationships between disease outbreaks and potential underlying processes when overlaying exposure and health outcome data on a single map. In emergency settings, the ability to visualize these spatiotemporal relationships between exposure events and disease outbreak signatures can help inform the timing and type of emergency interventions needed such as food aid delivery, natural disaster relief packages, or peacekeeping operations.

**Fig. 1:**
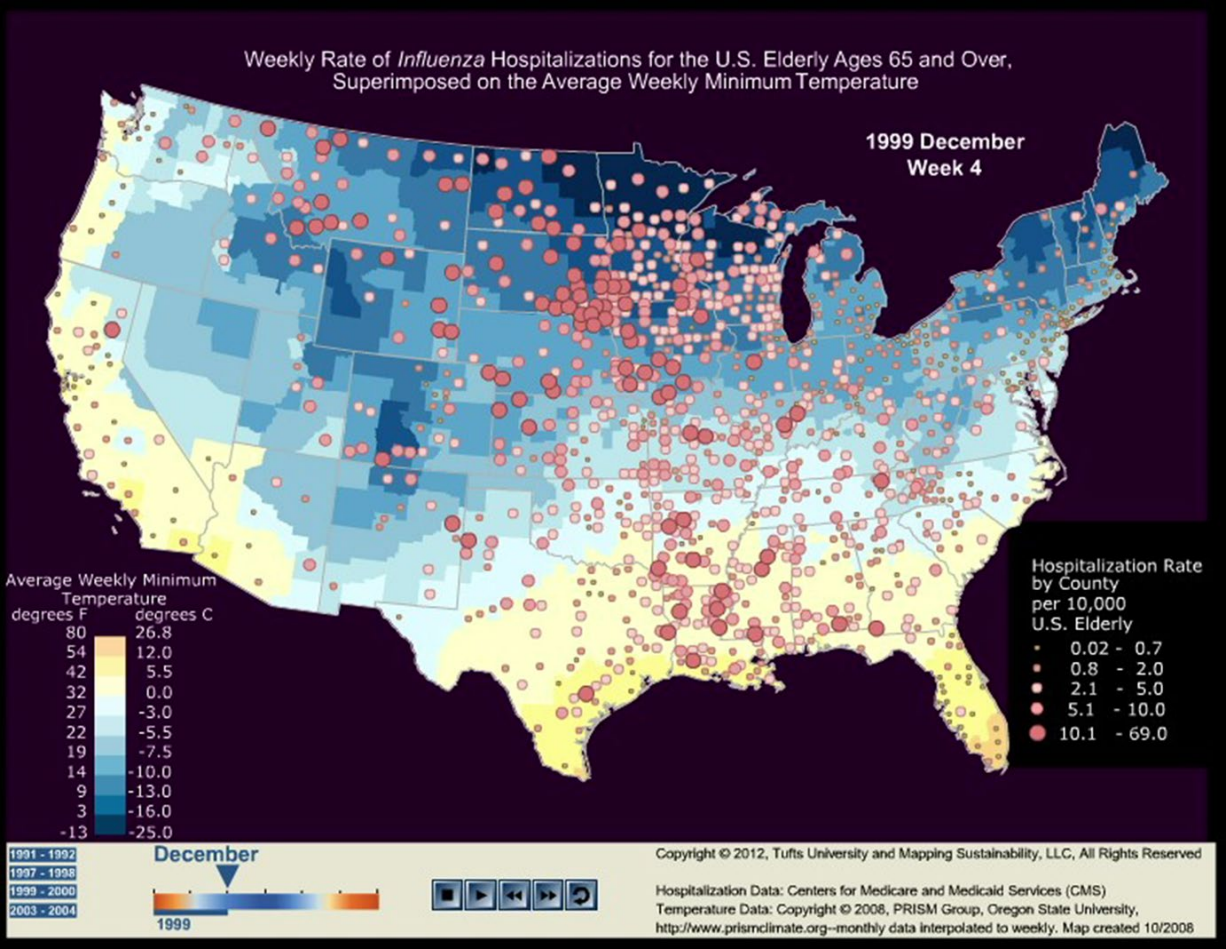
A snapshot of Movie 1, a moving set of images showing weekly rates of elderly (aged ≥ 65 years) influenza hospitalizations per 10,000 persons and average weekly minimum temperature in the United States for the 1991–1992, 1997–1998, 1999–2000, and 2003–2004 influenza seasons [27]. The size and darkness of red colour indicates more intense influenza outbreaks. Weekly minimum temperatures ranged from − 25 °C (− 13°F, dark blue colour) to ~  + 27 °C (+ 80°F, dark orange colour). The superimposition of weekly minimum temperature and elderly hospitalization rates illustrates travelling waves of infection in the United States and their association to fluctuations in temperature. To watch and download Movie 1, please see the 10.1057/s41271-022-00345-x


In our prior work, we outlined key principles for consideration when creating dynamic maps and ways to explore them [25]. We also applied dynamic maps to evaluate persistent clusters and travelling waves of influenza in the United States’ elderly population for four influenza seasons (Fig. 1; Movie 1) [27]. This series of dynamic maps demonstrated the spatial heterogeneity and emergence of seasonal influenza outbreaks. Understanding factors that drive complex infectious disease dynamics allows for more targeted response recommendations to effectively control seasonal outbreaks or epidemics [27].

A dynamic movie showing weekly rates of elderly hospitalizations due to influenza and average weekly minimum temperature in the United States for the 1991–92, 1997–98, 1999–2000, and 2003–04 influenza seasons [24]. We extracted weekly rates of influenza hospitalizations in persons aged ≥ 65 years from the Centers for Medicare and Medicaid Services (CMS) from Week 1 of 1991 through Week 52 of 2004. Hospitalization rates per 10,000 persons are depicted at the county level where the size and darkness of red colour indicates more intense influenza outbreaks. We extracted weekly minimum temperature data from the PRISM Group at Oregon State University for this 13-year study. Weekly minimum temperatures range from −13˚F (dark blue colour) to +80°F (dark orange colour). The superimposition of weekly minimum temperature and elderly hospitalization rates illustrates traveling waves of infection in the United States and their association to fluctuations in temperature. A snapshot of this movie can be found in Figure 1.

In this study, we demonstrated the process of creating a weekly time series dataset that integrated data streams of Yemeni conflict events and fatalities with confirmed cholera infections and rates in 2016–2019. We used the WHO Eastern Mediterranean Regional Office’s (EMRO) daily, weekly, and monthly bulletins following the first confirmed cholera case in Sana’a on 1 October 2016 [29]. Our comprehensive dataset allowed for examination of persistent clusters and travelling waves of cholera outbreaks and conflict events. We included visualizing cholera outbreaks and conflict events in complex emergency settings. Dynamic maps allowed us to investigate when outbreaks and conflict events occurred, how long they persisted, how they differed across geographic locations, and the utility and usability of harmonized global health datasets in public health applications. Our findings can also help identify geographic hotspots experiencing both intense cholera outbreaks and persistent conflict events, and thus most in need of cholera reduction strategies such as restoring infrastructure, improving water sanitation, and increasing access to cholera vaccines. With advanced technological platforms to support big datasets and animated maps, the global health community can move forward solutions to improve open-source surveillance systems and inform targeted health policies.

## Data and methods

### Conflict fatalities and events

We extracted daily conflict fatalities and events from ACLED reports [30]. ACLED partners with the Yemen Data Project (YDP) to collate and disseminate data on Yemeni war conflicts to improve transparency and promote actor accountability [30]. ACLED cross-references reports with daily and weekly Arabic and English news articles from 450 local, national, and international media agencies [30, 31]. ACLED reported on violent (for example, battles, explosions, violence against civilians) and non-violent (for example, protests, riots, strategic developments) political events, and estimated fatalities when possible [32, 33]. Reports cover ~ 70% of all urban and remote locations within Yemen. However, Houthi- and other warring party-controlled areas strongly censored conflict and political information, resulting in lesser coverage for Al-Jawf and Hajjah governorates [34, 35]. ACLED reported conflict events as discrete, daily, geocoded observations that repeated if multiple events occurred on the same day [34, 35]. We extracted daily records from 4 January 2016 through 29 December 2019, summed all-cause fatalities and events per day, and aggregated daily counts by week and governorate (0% missing data).

### Compilation of weekly cholera records

We extracted confirmed cholera infections from the WHO EMRO epidemiological bulletins for all Yemeni governorates. However, analysed only 20 of 21 governorates, as the WHO did not consistently report records for the Hadhramaut governorate within our study period. The EMRO began publishing these reports on 04 September; no data were available for the first 35 calendar weeks of 2016 [29, 36–38]. We assumed weekly counts of 0 infections for these weeks. We extracted, aggregated, and interpolated all reported daily, weekly, and monthly data from Week 36 of 2016 through Week 52 of 2019. To harmonize temporal and spatial resolution into a final curated dataset, we aggregated data by WHO-defined epidemiological weeks, an international standard defining weeks from Monday through Sunday starting with the full week of the year [18, 39].

Daily data from Weeks 36–45 of 2016 included cumulative counts of confirmed cholera infections for each governorate. However, EMRO epidemiological bulletins reported infections nationally from Week 46 of 2016 to Week 12 of 2017. For both time periods, we estimated average daily infections by subtracting cumulative totals from consecutive reports and dividing by the multi-day reporting length. We did not round fractional daily estimates to best estimate the distribution of infections in the absence of consistently-reported daily records. We distributed national daily average infections across governorates according to their relative population sizes using 2017 estimates, then aggregated daily averages by week [40]. While the spatial distribution of infections was likely a function of more than just population size, we found no additional data on risk factors like hygiene conditions or population displacement for estimating governorate-level case distributions. We assumed relative differences in population size adequately reflected relative differences in the transmissibility of incident infections. The WHO reported only 112 infections nationally during this 18-week period.

As the epidemic progressed, the WHO revised the EMRO epidemiological bulletin into weekly situation reports. These reports provided records of weekly cholera infections and deaths by governorate [38]. From Week 29 of 2017 to Week 26 of 2018, the WHO used a standardized reporting format that provided records for the week of publication and 3 weeks of historic records prior to the publication week (0–3-week lags). With each successive report, the WHO revised records within prior weeks to provide more accurate estimates of cholera infections adjusted for testing and reporting delays. For our analysis, we compiled the most updated records of confirmed case estimates available from each report.

Situation reports transitioned from weekly to monthly temporal aggregation in mid-2018. We extracted monthly data from Week 27 of 2018 through Week 52 of 2019. These reports provided cumulative confirmed infections per governorate from 27 April 2017 until the last day of the reporting month. We subtracted monthly totals of cholera infections from consecutive reports to estimate the cumulative total incidence of confirmed cholera infections. We divided this total by the number of days within that month to approximate average daily counts. Though daily reported infections are expected to fluctuate over a month’s time, no data with greater temporal granularity were available. We aggregated daily estimates by WHO-defined weeks to estimate weekly counts.

Irregular WHO bulletin-reporting frequency resulted in numerous weeks with missing records, or a time point when we could not find confirmed cholera case information. These reporting gaps occurred when the WHO transitioned from daily to weekly or weekly to monthly reporting formats. We had missing data for Weeks 13–16 of 2017, Week 8 of 2018, and Weeks 27–30 of 2018. To enhance the usability of our curated dataset, we handled missing data in two ways. First, we created a variable of reported infections that preserved missing data to allow data users to interpolate data if and how they may wish. Second, we created a variable with interpolated missing records using a linear approximation of reported adjacent weeks, as the most conservative representation of the epidemic curve.

### Calculating cholera rates

We created a weekly time series of population estimates, adjusted for population growth and conflict fatalities, to calculate weekly cholera rates [18]. First, we calculated each governorate’s population in Week 1 of 2017 using the average from multiple sources [38, 40, 41]. Next, we prorated an annual birth rate (≈ 0.024) from the 2004 Yemeni Population Census to reflect the low-fertility and moderate-mortality expected during the Yemeni Civil War [40]. We found no additional information on population growth rate in the 2014 Yemeni Census [42]. For weeks preceding Week 1 of 2017, we added conflict fatalities and divided the sum by the annual prorated growth rate. For weeks succeeding Week 1 of 2017, we subtracted conflict fatalities and multiplied the difference by the annual prorated growth rate.

We estimated national rates by summing infections for all governorates and dividing by the summed weekly population estimates. We report cholera rates per 100,000 persons (abbreviated as ‘cph’), calculated by dividing weekly infections by population estimates with a multiplier of 100,000.

### Examining persistent outbreak and conflict clusters

We examined persistent clusters of cholera rates and conflict events using Spearman autocorrelations. We assessed autocorrelations for all available lags in our time series (207 weeks) where high autocorrelation values suggested strong temporal trends. We determined governorates with persistent clusters by identifying long streaks of consecutively strong (*ρ* > 0.70) and moderate (*ρ* > 0.40) lagged positive autocorrelations using heatmaps. We defined significant correlations as *α* < 0.05.

### Metadata reporting and software usage

We share metadata to provide information needed to replicate the process of data curation and to understand the benefits and limitations of this data upon reuse. Metadata consist of a codebook with variable names, definitions, value types, value units, coding schemes, and the original variable’s data sources. We performed all population prorating calculations using Excel (14.3.6) software and calculation equations are visible within the curated dataset. We provide epidemiological bulletins used to create cholera data and R code used for developing and exporting dynamic maps and figures on our *figshare* repository [39].

## Results

We created Movie 2 for cholera rates and Movie 3 for conflict events to illustrate the use of dynamic mapping for curated global health datasets. Movie 2 provides a dynamic map of governorate-level rates over the 208-week study period. With this dynamic map, we provided the desired data visualization experience using an optimized frame speed of 1 second per frame to give enough time for the viewer to identify clusters of infections, store this pattern into short-term memory, and compare older clusters to newer clusters when examining the following frame [25]. We also provided an interactive user interface to enable viewers to replay frames at their discretion using a sliding calendar bar that traced the national time series of cholera rates.


**Fig. 2:**
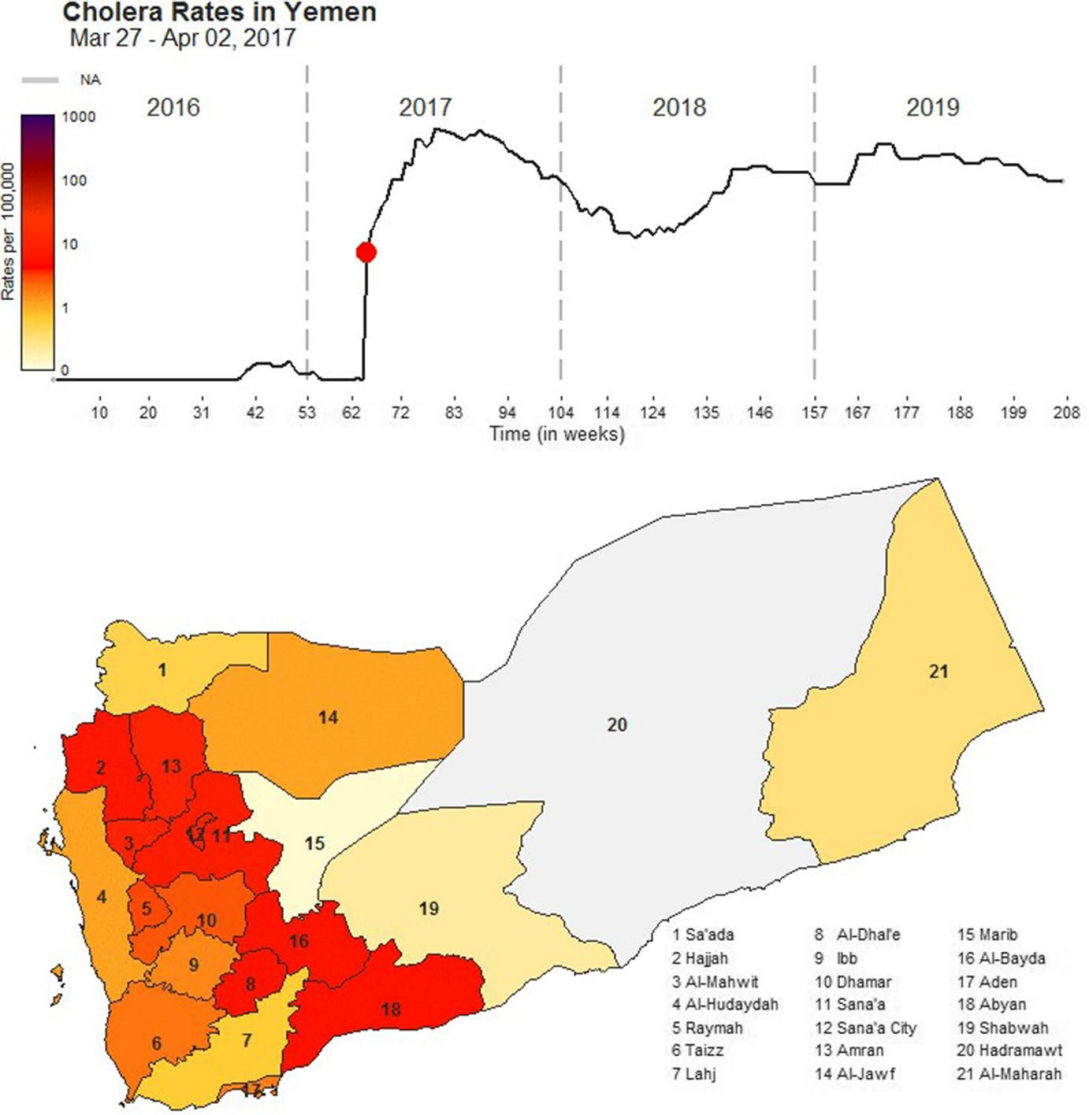
A snapshot of Movie 2, a dynamic movie showing weekly rates of confirmed cholera infections per 100,000 (cph) persons in 20 of 21 Yemeni governorates from Week 1 of 2016 through Week 52 of 2019 (208 weeks total). The top panel provides a time series of national cholera rates. Below, a governorate-level map illustrates the distribution of cholera rates per governorate. A light-yellow colour indicates rates of 0.00 cph while a deep purple colour indicates rates of 1000.00 cph. We used a logarithmic scale to properly correct the colour scheme for the variability of rates across governorates. We used a grey colour to indicate the Hadhramaut governorate for which no data were consistently reported for analysis. To watch and download Movie 2, please see the 10.1057/s41271-022-00345-x

A dynamic movie showing weekly rates of confirmed cholera infections per 100,000 (cph) persons in 20 of 21 Yemeni governorates from Week 1 of 2016 through Week 52 of 2019 (208 weeks total). The top panel provides a time series of national cholera rates. Below, a governorate-level map of the country illustrates the distribution of cholera rates per governorate. A light-yellow colour indicates rates of 0.00cph while a deep purple colour indicates rates of 1000.00cph. We used a logarithmic scale to properly correct the colour scheme for the variability of rates across governorates. We used a grey colour to indicate the Hadramaut governorate for which no data were consistently reported for analysis. A snapshot of this movie can be found in Figure 2

We standardized mapping properties to ease the examination of spatiotemporal disease dynamics including when outbreaks occurred, how long they persisted, and differences by governorate [25]. For example, we converted daily and monthly records into weekly estimates to reduce ‘noise’ and sporadic fluctuations typical for daily records while preventing the over-smoothing typical for monthly records. We applied a complimentary colour gradation to emphasize differences over time and location. As seen in Movie 2 (Fig. 2), infection rates varied from ~ 0.01 cph during outbreak nadirs to ~ 772.35 cph at outbreak peaks. We used a logarithmic transformation to extend the colour gradation evenly across this range. We selected complimentary colours (yellow and purple) of varying hues so that viewers with colour-blindness could distinguish variations in outbreak intensity.

Nationally, cholera rates had moderate-to-strong positive autocorrelation values for lags 1–14 (*p* < 0.001; Supplementary Figure S1). The length of these consistently moderate-to-strong correlations reflects the persistent national cholera outbreak throughout our study period. We used Movie 2 to identify possible travelling waves of infection from Sana’a, Sana’a City, Al-Mahwit, and Amran to surrounding areas. These governorates demonstrated high rates starting on 22–28 May 2017 (Supplementary Figure S2A). In the 14 weeks that followed (Supplementary Figures S2B-S2D), rates of infection increased rapidly in surrounding governorates suggesting a wave of infection outward to remote governorates of the country (Supplementary Figure S2). While Amran and Al-Mahwit had persistently high rates of infection, rates decreased for Sana’a and Sana’a City from 19–25 June to 17–23 July 2017. At the same time, cholera rates increased for Al-Dhale’e, Dhamar, Al-Bayda, Lahj, and Abyan, which all reached near-peak rates in 21–27 August 2017.

We also used Movie 2 to identify persistent clusters of high rates in Sa’ada, Sana’a, and Sana’a City throughout our study period (Supplementary Figure S3). These governorates had moderate-to-strong positive correlations for ~ 20 lags (*p* < 0.001) indicating persistent temporal trends. While cholera rates remained moderate in Sa’ada, rates were persistently high in Sana’a and Sana’a City even when national rates decreased to a local minimum in 16–22 April 2018 (Supplementary Figure S3A). When national rates reached a third and fourth local maximum in 01–07 October 2018 and 08–14 April 2019, rates remained elevated in Sana’a and Sana’a City compared to other governorates (Supplementary Figures S3B–S3C). Rates decreased little for these governorates by the conclusion of our study period (23–29 December 2019; Supplementary Figure S3D).

**Fig. 3:**
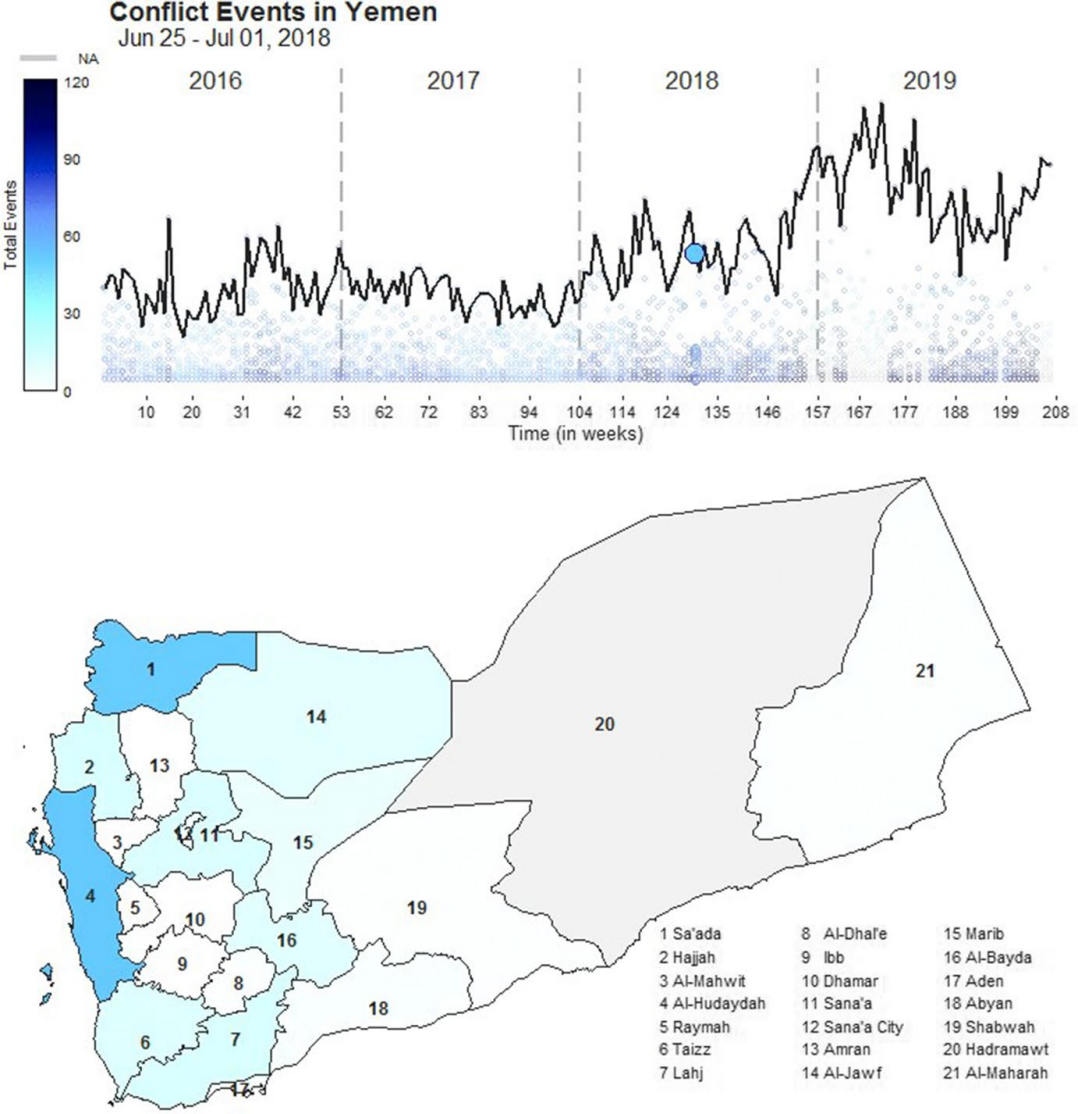
A snapshot of Movie 3, a dynamic movie showing the weekly sum of conflict events in 20 Yemeni governorates and nationally from Week 1 of 2016 through Week 52 of 2019. The top panel provides a bubble plot and time series of the national conflict events. Below, conflict event intensity is illustrated by governorate using a shaded map. A white colour indicates 0 events while a deep purple colour indicates 120 events. We selected the colour scheme to properly correct for the variability of conflict events across governorates. We used a grey colour to indicate the Hadhramaut governorate for which the WHO did not consistently report data for analysis. To watch and download Movie 3, please see 10.1057/s41271-022-00345-x


Movie 3 provided a dynamic map of governorate-level conflict events to show spatiotemporal fluctuations of exposure factors influencing cholera outbreaks (Fig. 3). We emphasized governorate-level variability in conflict events by providing a bubble plot below the time series line plot that reflects national conflict events. National conflict events had moderate-to-strong positive autocorrelation values for a 1–8-week lag period (p < 0.001; Supplementary Figure S4). This shorter lag interval compared to cholera rates illustrates the increased variability of conflict events during the Yemeni Civil War in 2016–2019. When inspecting autocorrelation values across governorates, we found that Al-Hudaydah had persistently moderate-to-strong positive autocorrelation values in lags 1–38 (*p* < 0.001). This illustrates a regional hotspot of conflict events clearly visualized using Movie 3. Al-Hudaydah conflict events rose sharply in June of 2018 (Supplementary Figure S5A) and accounted for nearly all events near and at the national peak in 2019 (Supplementary Figure S5B–S5C). Even as national events declined in October of 2019, Al-Hudaydah remained a persistent cluster of conflict events (Supplementary Figure S5D).

A dynamic movie showing the weekly sum of conflict events in the 20 of 21 Yemeni governorates for which we found data, and nationally from Week 1 of 2016 through Week 52 of 2019. The top panel provides a bubble plot and time series of the national conflict events. Below, we illustrate conflict event intensity by governorate using a shaded map. A white colour indicates 0 events while a deep purple colour indicates 120 events. We selected the colour scheme to properly correct for the variability of conflict events across governorates. We used a grey colour to indicate the Hadramaut governorate for which no data were consistently reported for analysis. A snapshot of this movie can be found in Figure 3

## Discussion

Our research demonstrated the importance of public data sharing using a standardized approach for extracting, aligning, and integrating spatiotemporal data. Our curated dataset enabled the creation of dynamic maps to investigate travelling waves and persistent clusters of infection rates and conflict events during the Yemeni Civil War in 2016–2019. Movie 2 illustrated a persistent cluster of rates in Sana’a and Sana’a City supported by moderate-to-strong positive autocorrelation values for a ~ 20-week lag period. This movie also allowed us to identify a possible travelling wave of infection from this cluster to surrounding governorates in May–August of 2017.

We found a shorter lag period of moderate-to-strong positive autocorrelation values nationally for conflict events reflecting greater variability of conflict events in most governorates. However, Al-Hudaydah demonstrated persistent correlations across ~ 40-week lag period. Movie 3 showed that Al-Hudaydah’s conflict events were greatest from June of 2018 through December of 2019. This increase coincided with assaults on Al-Hudaydah by pro-government forces, backed by the Saudi-led coalition, in June–November of 2018 [43]. These forces installed a blockade in Al-Hudaydah port, which continued to restrict both humanitarian medical supplies and food aid to the entire country the end of our study period [44].

Our findings illustrate the possibility of conducting early outbreak warnings if timely surveillance data are available and accessible. These efforts can help to develop humanitarian assistance strategies amidst ongoing public health emergencies. The cholera epidemic’s origin and persistence within Sana’a and Sana’a City suggests the importance of monitoring these governorates as markers for future outbreaks. Evidence of travelling waves from this epicentre necessitates the strengthening of health and environmental infrastructure and implementation of preventative infections’ mitigation strategies in surrounding governorates. These measures will reduce the likelihood of a national epidemic. Movie 3 also demonstrates the direct effect of war conflict in specific governorates, and therefore, challenges to implementing public health interventions. The persistent cluster of conflict events within Al-Hudaydah illustrates the extent of these challenges in complex emergency settings.

We encourage researchers to replicate dynamic mapping techniques for other data streams such as environment- or nutrition-related variables if properly aligned and integrated with our dataset. This will allow for data modellers to spatiotemporal associations between conflict-, environment-, or nutrition-related factors and cholera rates using our curated dataset. Many recent studies have explored factors associated with cholera transmission dynamics using granular remote sensing, climate-related spatial data, and conflict information [14, 45–51]. By improving data collection and processing capabilities, public health professionals will be well equipped with the data and tools to embrace a new era of precision health that prioritizes the sharing of granular temporal and spatial information and creation of high-quality data visualizations that capture complex spatiotemporal patterns of disease outbreaks. We recommend that future research use modelling approaches that properly account for complex, non-linear, and spatially-autocorrelated relationships between these variables.

Modern surveillance systems must improve to reflect both how internal data curators collect, store, monitor, and manage data––and how external data users extract, process, and analyse these data. Such systems can offer near- and real-time forecasts, long-term trend analyses, and outbreak modelling to develop early outbreak warnings and inform timely aid resource deployment. Disease surveillance systems should ensure data transparency and longevity by developing strong protocols for metadata standardization [52]. Improving data quality and availability corresponds with a greater need for prioritizing information management within and across national and international public health, environmental, and humanitarian emergency agencies and organizations. A lack of coordination in data collection and sharing reduces the availability of granular temporal and spatial data for public use. In turn, this forces efforts and decision-making to occur at coarser spatial and temporal scales, reducing the efficacy and refinement of public health and humanitarian interventions.

### Study limitations

From Week 46 of 2016 to Week 12 of 2017, we distributed national daily average cholera infections across governorates according to relative population estimates. We made this approximation by assuming that cholera outbreaks followed specific population transmission dynamics with higher incidence in more densely populated locations [53]. Additionally, we reported missing weeks only if all days within that week had a missing estimate. Though sensitive to underestimation, this approach maximized the utility of available surveillance data for conducting time series analyses in the absence of additional information to estimate weekly rates. We stress the need for national public health agencies, international health organizations, and the global health community at large to dedicate more resources and funds to implement thorough infectious disease outbreak investigations worldwide [54].

ACLED methodological codebooks noted that fatalities were not easily verified and prone to manipulation by armed groups [32]. Even so, these estimates provided the most accurate and reliable approximation of all-cause conflict fatalities during the Yemeni Civil War (neither civilian nor bystander causalities reported) [31]. Furthermore, ACLED and YDP validated all fatality and conflict event information using a combination of health reports, news articles, field surveys, and media stories [31, 32]. While population estimates fluctuate dramatically during conflict due to rapid internal displacement and external migration, we lacked sufficient temporally granular displacement and migration data to improve our adjusted population rate calculations [55].

We used a 2017 population estimate calculated as the average of the WHO EMRO epidemiological bulletins (from which we extracted cholera infections), Yemeni Central Statistical Organization (in-country reporting), and the International Organization of Migration’s Displacement Tracking Matrix (monitoring migration during humanitarian emergencies) reports [38, 40, 41]. Together, these estimates provided the best approximation for governorate-level population. We prorated weekly population estimates using a low-fertility and moderate-mortality birth rate estimate and ACLED fatalities to favour under-reporting of infection rates during the Yemeni Civil War [56]. We found no alternative calculation technique for describing population estimation during conflict events or humanitarian emergencies.

We used various data sources to harmonize this dataset including health reports, news articles, and field surveys. We believe these reports provided accessible, usable, and timely documentation of information related to Yemeni cholera infections and conflict-related outcomes in 2016–2019. While encouraged by this harmonization process, researchers must recognize that all estimates were only as accurate as the reports from which data were extracted. We strove for clarity and transparency in the applied methods, yet we acknowledge that metadata on the pre-processing of publicly available data from international organizations was extremely limited. Where possible, we compiled the metadata or raw text files for the extracted records used to create this dataset and uploaded files to our *figshare* repository [39].

### Future directions

The essence of an informative dynamic map is strong data structure and a rigorous process of compilation. In this study, we demonstrated that the curation of comprehensive global health repositories enabled the creation of dynamic maps for tracking, recognizing, and visualizing complex spatiotemporal processes. The standardization and harmonization of reporting publicly available data ensures the longevity of data usability even as the platforms used to store, analyse, and communicate data change over time. Curated global health datasets and web-based dashboards with built-in dynamic mapping tools improve the reporting and understanding of associations between diseases and manmade or natural risk factors. Both dynamic maps and the process of data extraction, aggregation, and alignment emphasize the importance of long-term surveillance data collection in usable time series data formats. Only with these tools can surveillance records and dynamic mapping be used effectively and efficiently to plan for and respond to complex emergencies with medical, fiscal, and humanitarian supplies and aid resources [57].

Our data curation techniques can be applied to updated cholera data when it becomes publicly available for the Yemeni outbreak. Researchers can also apply our data extraction, alignment, and compilation techniques for other infectious disease outbreaks worldwide, as many WHO globally monitored infections share similar epidemiological bulletin reporting formats. We encourage researchers to harmonize and integrate more global public health data streams within this dataset, especially those from the WHO and World Food Programme (WFP) [58–63]. Research has documented how environmental risk factors amplify cholera infection rates at the district level, though few studies have explored these factors in combination with other conflict- or nutrition-related risk factors [13]. While key informant interviews have noted increased cholera morbidity and mortality in individuals with poor nutrition status, we found no studies investigating temporal relationships between cholera infection rates and risk factors related to food access, purchasing power, or insecurity [2, 3, 64]. Future research must be translational; we encourage researchers to standardize and harmonize data at granular spatial scales to inform and empower local actors to promote public health programming under constrained resource circumstances.

We reported confirmed cholera infections as weekly time series in two ways: with and without interpolating missing data. We urge the global health community to standardize reporting of missing with respect to reasons for, quantity of, and location where missing data occur within a time series. Metadata reports can include information describing the completeness of time series data over time and by geographic location [52]. These reports inform data users how interpolation techniques impact time series analyses and forecasts [65]. Our attempts at interpolating missing data demonstrated the difficulties and ambiguity of using publicly reported time-referenced data when no metadata or standardized reporting protocol exists. These concerns and difficulties also occurred when extracting and aligning conflict-related time series data.

## Conclusions

The global public health community needs comprehensive interdisciplinary data repositories and platforms supported by standardized data collection protocols. Modern surveillance systems should seek to harmonize the spatial and temporal resolution of time-referenced data so external users can effectively trace the onset, spread, and amplification of infections. We implore data-curating organizations to collect public health records in a way that ensures their long-term usability and should be treated as public health investments. We demonstrated how to curate public records in this way and used dynamic maps to trace the onset and spread of infection and conflict events that amplified the cholera outbreak in Yemen. Our dynamic maps suggest travelling waves of infection from Sana’a and Sana’a City to surrounding governorates as well as a persistent cluster of conflict events in Al-Hudaydah from June of 2018 through December of 2019. Future research must integrate additional global health data streams and utilize this visualization technique to uncover more associations between the cholera outbreaks and conflict-, environment-, and nutrition-related risk factors driving infection.

## Supplementary Information

Below are the links to the electronic supplementary materials.

Supplementary file4 (DOCX 1081 kb)
