# Supplementary material for: Dynamic mapping of cholera outbreak during the Yemeni Civil War, 2016–2019
Source: J Public Health Policy. 2022 May 25;43(2):185–202. doi: 10.1057/s41271-022-00345-x (PMC9192410; doi:10.1057/s41271-022-00345-x)
Supplement: Supplementary file 4 — Supplementary file4 (DOCX 1081 kb) [file 41271_2022_345_MOESM4_ESM.docx]

**Journal of Public Health Policy**

Supplementary Materials

Dynamic mapping of cholera outbreak during the Yemeni Civil War, 2016-2019

Ryan B. Simpson ^1,^*, Sofia Babool ^2^, Maia C. Tarnas ^3^, Paulina M. Kaminski ^1^, Meghan A. Hartwick ^1^, Elena N. Naumova ^1,^*

1. Nutrition Epidemiology and Data Science Department, Tufts University Friedman School of Nutrition Science and Policy, Boston, MA, USA
2. Neuroscience Department, The University of Texas at Dallas, Richardson, TX, USA
3. Community Health Department, Tufts University School of Arts and Sciences, Medford, MA, USA

**Corresponding Authors:**

Elena N. Naumova

[elena.naumova@tufts.edu](mailto:elena.naumova@tufts.edu)

150 Harrison Avenue, Boston, MA 02111

Ryan B. Simpson

[ryan.simpson@tufts.edu](mailto:ryan.simpson@tufts.edu)

150 Harrison Avenue, Boston, MA 02111

**Running Title:**

Dynamic mapping of Yemeni cholera outbreak

**Journal of Public Health Policy**

Supplementary Materials

Dynamic mapping of cholera outbreak during the Yemeni Civil War, 2016-2019

**Movie 1.** A dynamic movie showing weekly rates of elderly hospitalizations due to influenza and average weekly minimum temperature in the United States for the 1991-92, 1997-98, 1999-2000, and 2003-04 influenza seasons [24]. We extracted weekly rates of influenza hospitalizations in persons aged ≥65 years from the Centers for Medicare and Medicaid Services (CMS) from Week 1 of 1991 through Week 52 of 2004. Hospitalization rates per 10,000 persons are depicted at the county level where the size and darkness of red colour indicates more intense influenza outbreaks. We extracted weekly minimum temperature data from the PRISM Group at Oregon State University for this 13-year study. Weekly minimum temperatures range from -13˚F (dark blue colour) to +80˚F (dark orange colour). The superimposition of weekly minimum temperature and elderly hospitalization rates illustrates traveling waves of infection in the United States and their association to fluctuations in temperature. A snapshot of this movie can be found in Figure 1.

**Movie 2.** A dynamic movie showing weekly rates of confirmed cholera infections per 100,000 (cph) persons in 20 of 21 Yemeni governorates from Week 1 of 2016 through Week 52 of 2019 (208 weeks total). The top panel provides a time series of national cholera rates. Below, a governorate-level map of the country illustrates the distribution of cholera rates per governorate. A light-yellow colour indicates rates of 0.00cph while a deep purple colour indicates rates of 1000.00cph. We used a logarithmic scale to properly correct the colour scheme for the variability of rates across governorates. We used a grey colour to indicate the Hadramaut governorate for which no data were consistently reported for analysis. A snapshot of this movie can be found in Figure 2.

**Movie 3.** A dynamic movie showing the weekly sum of conflict events in the 20 of 21 Yemeni governorates for which we found data, and nationally from Week 1 of 2016 through Week 52 of 2019. The top panel provides a bubble plot and time series of the national conflict events. Below, we illustrate conflict event intensity by governorate using a shaded map. A white colour indicates 0 events while a deep purple colour indicates 120 events. We selected the colour scheme to properly correct for the variability of conflict events across governorates. We used a grey colour to indicate the Hadramaut governorate for which no data were consistently reported for analysis. A snapshot of this movie can be found in Figure 3.

**All movies can be found at:** <https://tufts.box.com/v/Simpson-2022-CholeraDynamicMap>


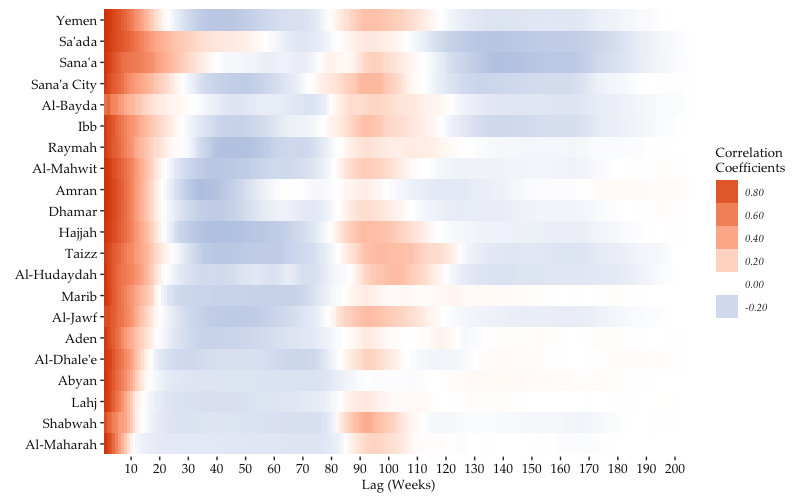


**Supplementary Figure S1.** A heatmap of autocorrelation coefficient values for weekly rates of confirmed cholera infections per 100,000 (cph) persons in 20 Yemeni governorates and nationally. A dark orange colour indicates strong positive autocorrelations (ρ > 0.80), a light blue colour indicates moderate negative autocorrelations (ρ < -0.20), and a white colour indicates no correlation (ρ = 0.00). Time is reported in lagged weeks and is estimated for all 207 lags permitted within our 208-week time series. We order governorates according to the number of consecutive lagged weeks with consistently significant strong-to-moderate positive autocorrelation values (i.e., ρ > 0.80, p<0.05).


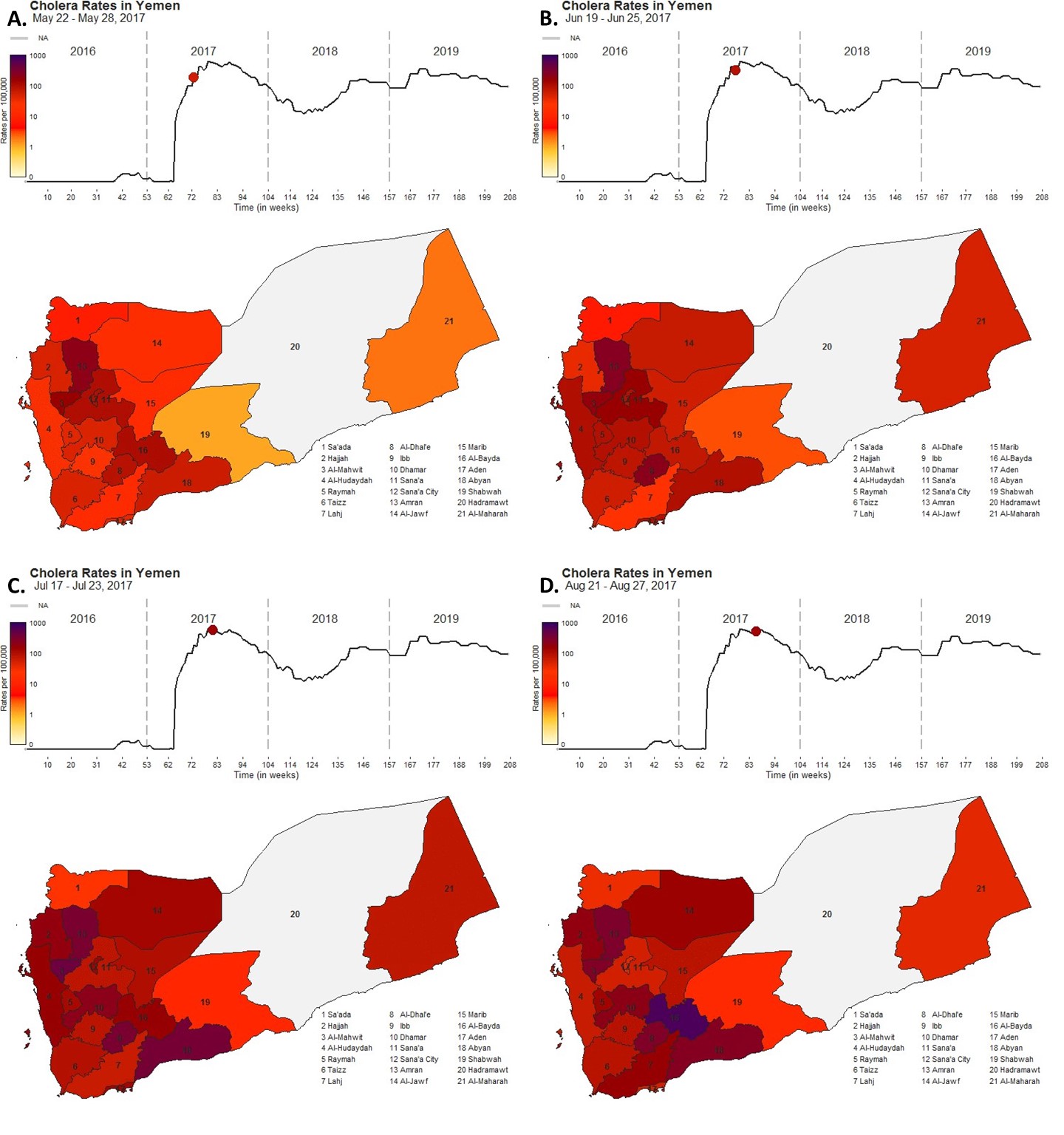


**Supplementary Figure S2.** Four weeks from Movie 2 showing weekly cholera rates per 100,000 (cph) persons in 20 Yemeni governorates and nationally. Time points include: (A) 22-28 May 2017, (B) 19-25 June 2017, (C) 17-23 July 2017, and (D) 21-27 August 2017. We selected these dates to illustrate the percolation of the cholera outbreak from governorates surrounding the nation’s capital (Sana’a, Sana’a City, Al-Mahwit, Amran) to neighbouring governorates. A light-yellow colour indicates rates of 0.00cph while a deep purple colour indicates rates of 1000.00cph. We used a logarithmic scale to properly correct the colour scheme for the variability of rates across governorates. We used a grey colour to indicate the Hadramaut governorate for which no data were consistently reported for analysis.


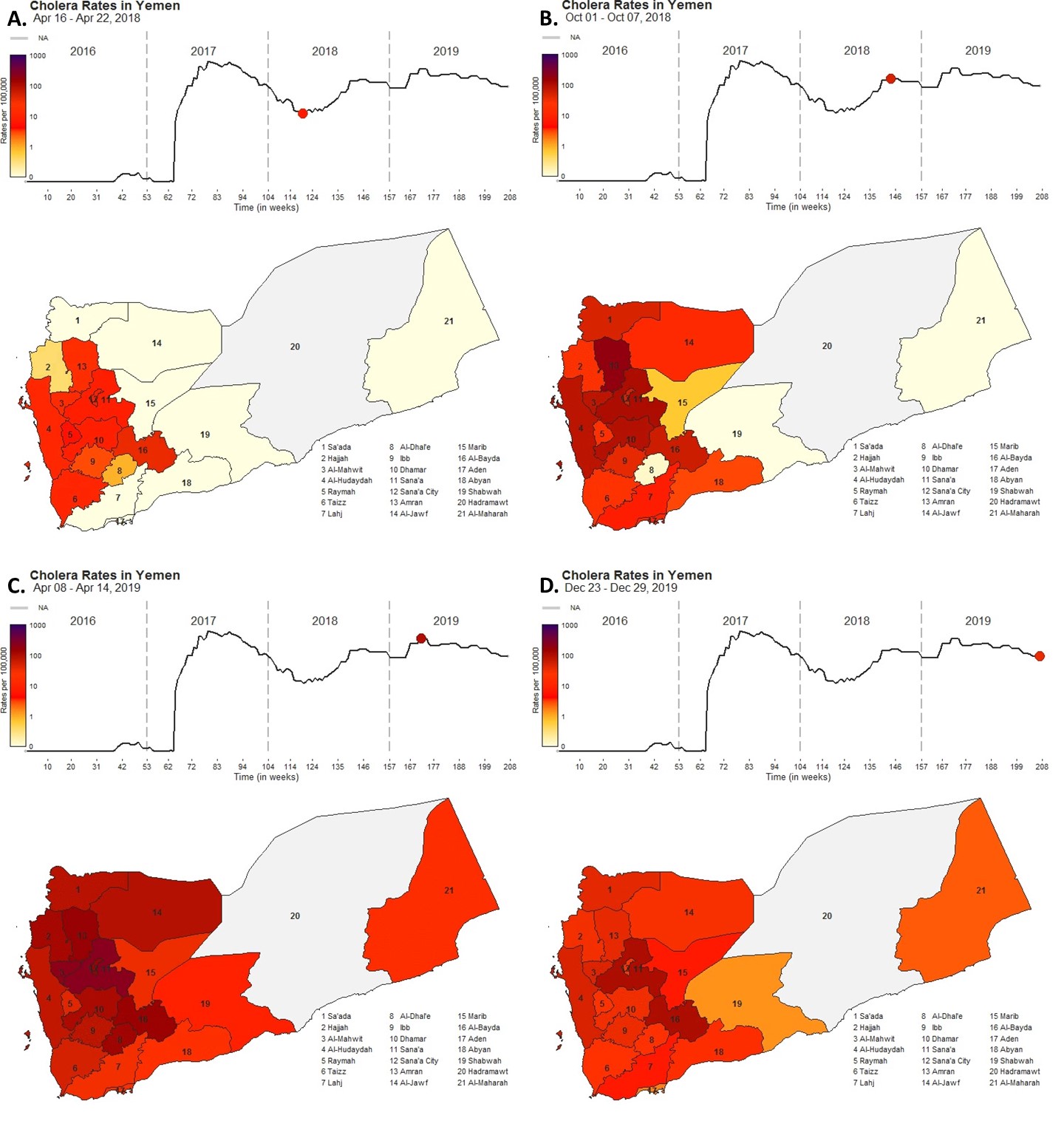


**Supplementary Figure S3.** Four weeks from Movie 2 showing weekly cholera rates per 100,000 (cph) persons in 20 Yemeni governorates and nationally. Time points include: (A) 16-22 April 2018, (B) 01-07 October 2018, (C) 08-14 April 2019, and (D) 23-29 December 2019. We selected these dates to illustrate the persistent cluster of high cholera rates in Sana’a and Sana’a City. A light-yellow colour indicates rates of 0.00cph while a deep purple colour indicates rates of 1000.00cph. We used a logarithmic scale to properly correct the colour scheme for the variability of rates across governorates. We used a grey colour to indicate the Hadramaut governorate for which no data were consistently reported for analysis.


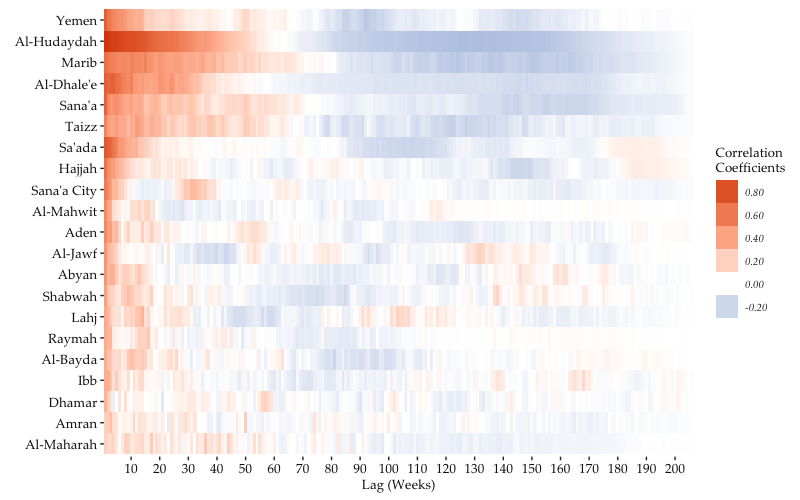


**Supplementary Figure S4.** A heatmap of autocorrelation coefficient values for all-cause conflict events in 20 Yemeni governorates and nationally. A dark orange colour indicates strong positive autocorrelations (ρ > 0.80), a light blue colour indicates moderate negative autocorrelations (ρ < -0.20), and a white colour indicates no correlation (ρ = 0.00). We reported time in lagged weeks and estimated this for all 207 lags permitted within our 208-week time series. We order governorates according to the number of consecutive lagged weeks with consistently significant strong-to-moderate positive autocorrelation values (i.e., ρ > 0.80, p<0.05).


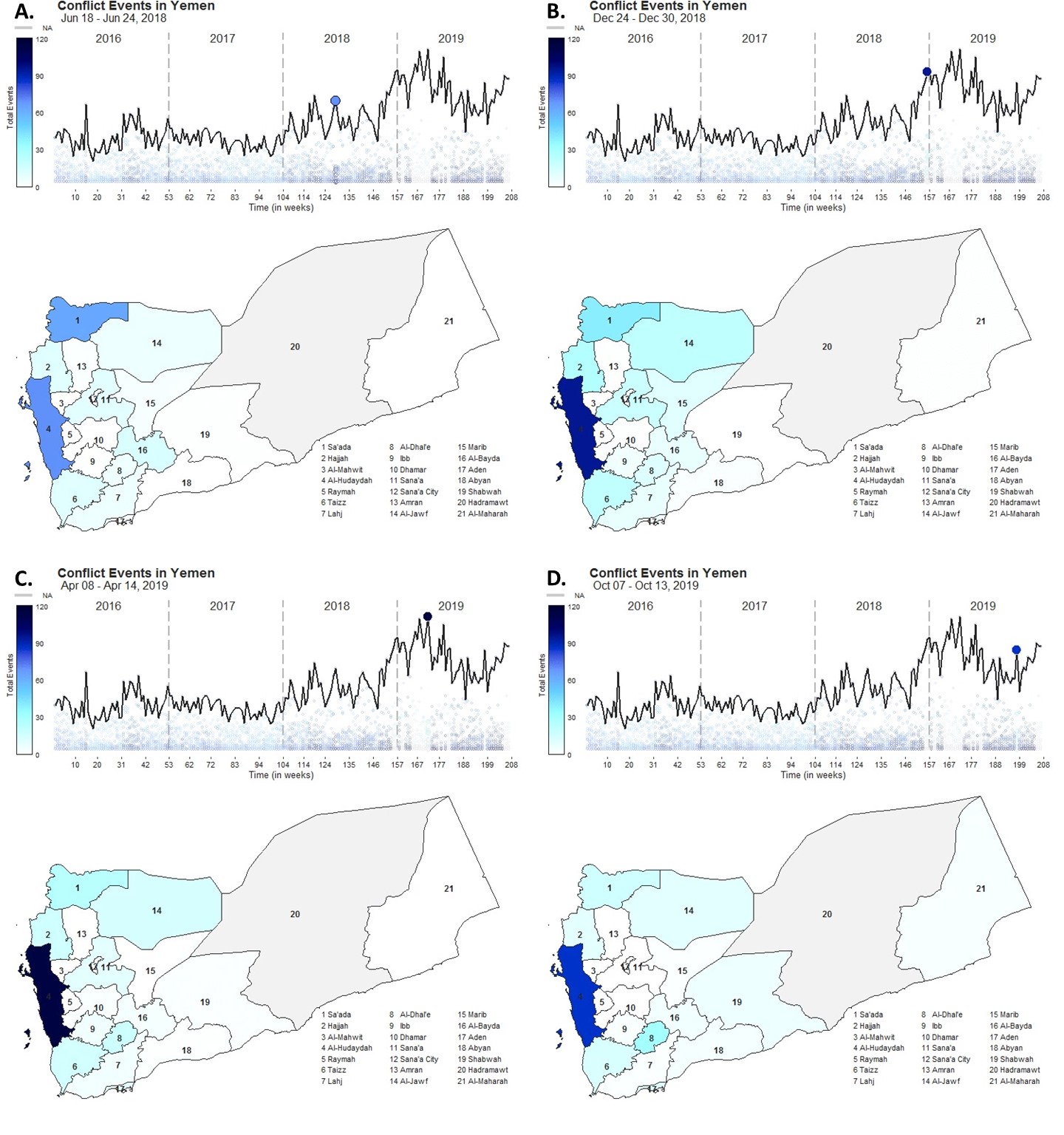


**Supplementary Figure S5.** Four weeks from Movie 3 showing weekly cumulative totals of all-cause conflict events in 20 Yemeni governorates and nationally. Time points include: (A) 18-24 June 2018, (B) 24-30 December 2018, (C) 08-14 April 2019, and (D) 07-13 October 2019. We selected these dates to illustrate the regional hotspot of conflict events in Al-Hudaydah from June of 2018 through October of 2019. A white colour indicates 0 events while a deep purple colour indicates 120 events. We selected the colour scheme to properly correct for the variability of conflict events across governorates. We used a grey colour to indicate the Hadramaut governorate for which no data were consistently reported for analysis.
